# The Morphia Habit

**Published:** 1892-03

**Authors:** 


### THE MORPHIA HABIT.

### A VICE WHICH HAS COME TO BE VERY COMMON IN PARIS.

The London *Standard's* Paris correspondent makes some revelations as to the wide prevalence in that capital of the use of morphia. The correspondent says that the habit of using hypodermic injections of morphia has spread, and is still spreading, with alarming rapidity.

Some years ago there were a certain number of doctors who carried on a proselytizing campaign in favor of combating every sort of pain by morphia. I have been assured, from what I

believe to be a thoroughly trustworthy source, that one or two of these doctors went so far as to give dainty syringes inclosed in little morocco leather cases, to several of their patients. I cannot say whether this was frequently done, but a lady of my acquaintance showed me one day a morphia syringe which her doctor, a well-known French practitioner, had given her. This little present was, of course, accompanied with a prescription for the morphia.

Those tiny bright instruments, which were, and are still to be had at almost every chemist's shop, have had a considerable share in the rapid spread of the morphia habit here. They took the fancy of most people to whom they were given or sold. The possessors of them carried them about in their pockets, showed them to their friends, and, like all those addicted to morphia, did their utmost to make proselytes. I have used the past tense, but I am sorry to say the same thing is going on to-day, and it is a curious fact that there is no class of persons in Paris more addicted to the vice of morphia-taking than doctors and chemists, and their wives and families. In any case the number of habitual morphia-takers is now so large that it is affirmed there are several establishments in Paris where the poison is clandestinely administered.

Those debauches are naturally attended almost exclusively by young gentlemen, though some ladies who would call themselves respectable have been known to accept invitations to them. I have been informed by a person who was present at a morphia tea-party, given at the house of a demi-mondaine living in a street close to the Champs Elysees, that everything was conducted in the most decorous manner. The hostess received her guests with becoming courtesy, and while they were sipping cups of tea and munching biscuits, she administered to them the desired hypodermic injection.

Several of the guests were down-spirited on their arrival, but almost immediately after the little syringe had done its work they became animated, and even brilliant, in their conversation. The terrible reaction naturally followed, but it was not till they had left the gay company, and were brought face to face with realities in their own homes. That reaction from high spirits to a downcast humor would not probably be so dangerous if it were not attended with an irresistible craving for another dose of the poison. According to Dr. Oscar Jennings, who is authority on the subject, the craving arises from morphia being a poison for which the only antidote is morphia.

